# Self-assembly of tetrareduced corannulene with mixed Li–Rb clusters: dynamic transformations, unique structures and record ^7^Li NMR shifts†
†Electronic supplementary information (ESI) available: Experimental and synthetic procedures, characterization data, computational details, and crystallographic methods employed in this work are given. CCDC 1032187 and 1032188. For ESI and crystallographic data in CIF or other electronic format see DOI: 10.1039/c4sc03485f



**DOI:** 10.1039/c4sc03485f

**Published:** 2014-12-16

**Authors:** Alexander S. Filatov, Sarah N. Spisak, Alexander V. Zabula, James McNeely, Andrey Yu. Rogachev, Marina A. Petrukhina

**Affiliations:** a Department of Chemistry , University at Albany , State University of New York , Albany , NY 12222 , USA . Email: mpetrukhina@albany.edu ; Fax: +1-518-442-3462; b Department of Chemistry , University of Wisconsin , Madison , WI 53706 , USA; c Department of Biological and Chemical Sciences , Illinois Institute of Technology , Chicago , IL 60616 , USA

## Abstract

Self-assembly processes of the highly reduced bowl-shaped corannulene generated by the chemical reduction with a binary combination of alkali metals, namely Li–Rb, have been investigated by variable-temperature ^1^H and ^7^Li NMR spectroscopy. The formation of several unique mixed metal sandwich products based on tetrareduced corannulene, C_20_H_10_^4–^ (**1** ^4–^), has been revealed followed by investigation of their dynamic transformations in solutions. Analysis of NMR data allowed to propose the mechanism of stepwise alkali metal substitution as well as to identify experimental conditions for the isolation of intermediate and final supramolecular products. As a result, two new triple-decker aggregates with a mixed Li–Rb core, [{Rb(THF)_2_}_2_]//[Li_3_Rb_2_(C_20_H_10_)_2_{Li^+^(THF)}] (**2**) and [{Rb(diglyme)}_2_]//[Li_3_Rb_3_(C_20_H_10_)_2_(diglyme)_2_]·0.5THF (**3**·0.5THF), have been crystallized and structurally characterized. The Li_3_Rb_2_-product has an open coordination site at the sandwich periphery and thus is considered transient on the way to the Li_3_Rb_3_-sandwich having the maximized intercalated alkali metal content. Next, the formation of the LiRb_5_ self-assembly with **1** ^4–^ has been identified by ^7^Li NMR as the final step in a series of dynamic transformations in this system. This product was also isolated and crystallographically characterized to confirm the LiRb_5_ core. Notably, all sandwiches have their central cavities, located in between the hub-sites of two C_20_H_10_^4–^ decks, occupied by an internal Li^+^ ion which exhibits the record high negative shift (ranging from –21 to –25 ppm) in ^7^Li NMR spectra. The isolation of three novel aggregates having different Li–Rb core compositions allowed us to look into the origin of the unusual ^7^Li NMR shifts at the molecular level. The discussion of formation mechanisms, dynamic transformations as well as unique electronic structures of these remarkable mixed alkali metal organometallic self-assemblies is provided and supported by DFT calculations.

## Abstract 2

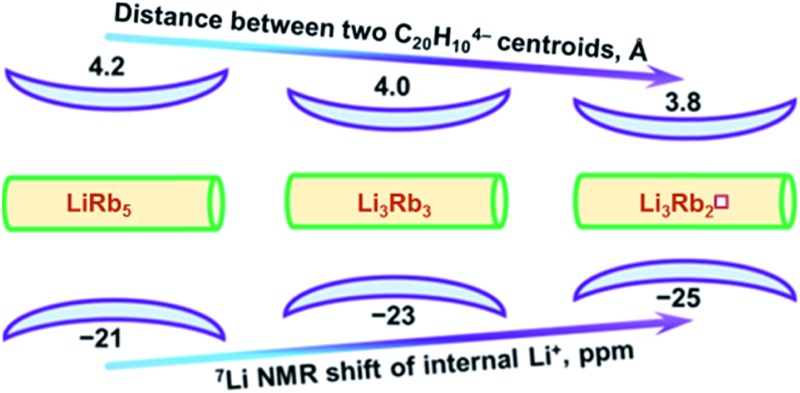
The record NMR shifts for the centrally encapsulated lithium ions are correlated with molecular and electronic structures of novel mixed alkali metal sandwiches.

## Introduction

**Scheme 1:**
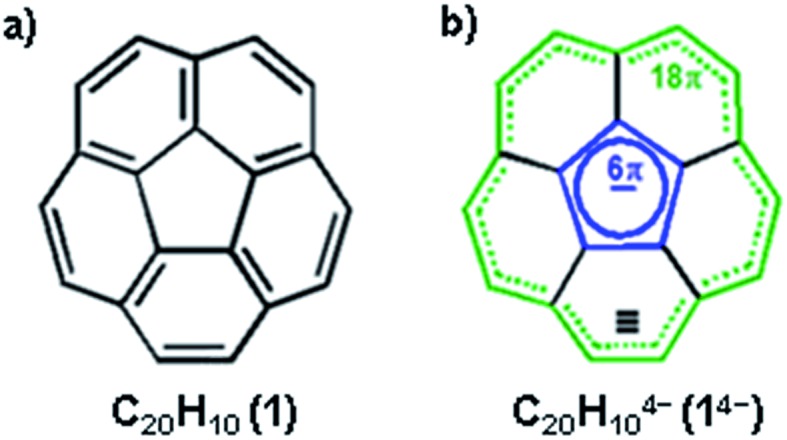
Corannulene (a) along with the electronic structure of C_20_H_10_^4–^ (b).

Non-planar carbonaceous aromatic compounds, such as fullerenes and carbon nanotubes, have recently emerged as promising light-weight materials for electronics and energy-related applications.^1^ Their potential application in lithium-ion battery technology has also attracted special attention.^2^ Moreover, the unique thermoelectric and superconducting properties of the reduced forms of curved carbon frameworks has triggered a renewed interest in these materials.^3^ All these recent advances stimulated a rapid expansion of the family of curved carbon-rich compounds which now includes a great variety of carbon bowls,^4^ warped nanographene sheets,^5^ as well as nanobelts and nanoribbons of different sizes.^6^ Bowl-shaped polyaromatic hydrocarbons (also referred to as carbon or π-bowls) have been broadly investigated over the last two decades to reveal their unique coordination and redox properties.^4,7^ For example, they can readily accept multiple electrons in stepwise reduction reactions to form the sets of the consequently reduced non-planar polyaromatic carbanions. The latter have been the subjects of numerous investigations^8^ due to their unique interplay of strain and conjugation. It was demonstrated that the smallest bowl-shaped polyarene, corannulene (C_20_H_10_, **1**, Scheme 1a) is able to undergo four reduction steps to form a set of corannulene anions, C_20_H_10_^*n*–^ (*n* = 1–4).^9^ The final four-fold reduction is accompanied by a significant core rearrangement of the corannulene tetraanion which can be considered to have an annulene-within-an-annulene electronic structure (Scheme 1b).^10^ Notably, C_20_H_10_^4–^ bears one electron per five carbon atoms and is more electron rich than the fullerene-hexaanion (one electron per ten carbon atoms in C_60_^6–^). This makes it a very interesting and unique carbanion for investigation of metal coordination and self-assembly reactions.

**Scheme 2:**
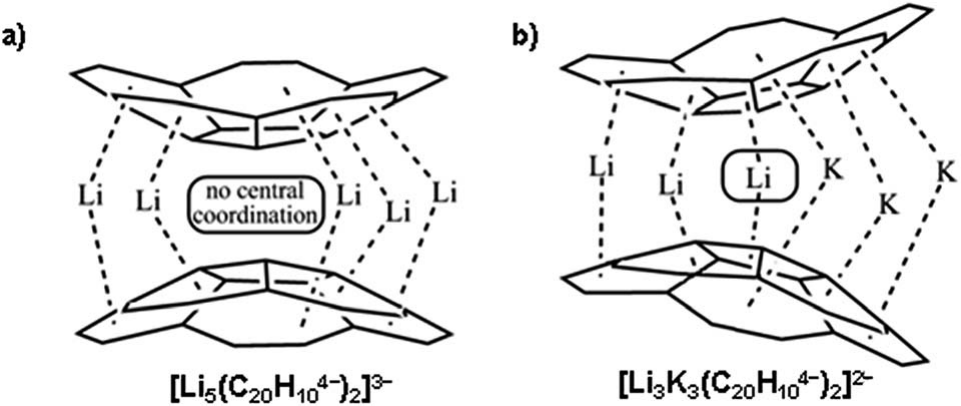
Supramolecular aggregates with (a) Li_5_- and (b) Li_3_K_3_-cores sandwiched between two C_20_H_10_^4–^ decks.

The highly reduced corannulene anions exhibit remarkable ability to form unique supramolecular aggregates in solutions, as shown by extensive ^1^H and ^7^Li NMR spectroscopy studies.^10^ However, structural investigations of the charged π-bowls were lagging behind their solution spectroscopic studies until we have accomplished the first X-ray diffraction characterization of the product formed by the tetrareduced corannulene with lithium counterions.^11^ The formation of a remarkable aggregate with a Li_5_-core sandwiched between two C_20_H_10_^4–^ decks has been established (Scheme 2a). This supramolecular aggregation with lithium ions allows to achieving a tetrareduced state of corannulene, as the electrochemical generation of C_20_H_10_^4–^ cannot be accomplished due to a very large negative standard potential located outside of the current experimental window.^12^ We have also demonstrated that the triple-decker supramolecular aggregate, [Li_5_(C_20_H_10_^4–^)_2_]^3–^, can be crystallized in different external coordination environments, showing no significant effect on the geometry of the sandwich core.^11,13^ In addition, the formation of the [Li_5_(C_20_H_10_^4–^)_2_]^3–^ product (abbreviated as Li_5_ below) is confirmed in solution based on the observed shifts for sandwiched Li^+^ ions (–11.70 ppm) and a proper 3 : 5 integration of external *vs.* internal ions in the ^7^Li NMR spectra. Importantly, these results illustrated the ability of tetrareduced corannulene to engage all of its adjacent six-membered rings in alkali metal binding and thus to encapsulate a large amount of Li^+^ ions, which can be related to the high charge capacity of the corannulene-based electrodes in Li-ion batteries.^14^

Remarkably, the coordination limit of the highly electron rich corannulene tetraanion can be further extended through the synergistic use of two alkali metals as the reducing agents. The concomitant reduction of C_20_H_10_ using a Li–K mixture resulted in the recent discovery of a novel class of mixed metal supramolecular products, in which tetrareduced corannulene exhibited its new coordination record.^15^ The C_20_H_10_^4–^ anion is able to engage all its sites, five benzene rings along with a central five-membered ring, for binding of six alkali metal ions in the resulting mixed metal core, Li_3_K_3_ (Scheme 2b) or LiK_5_, triple-decker sandwiches. Notably, the previously unseen involvement of the hub-site of corannulene tetraanion in lithium ion binding is accompanied by unprecedented negative shifts in ^7^Li NMR spectra (up to *ca.* –25 ppm).^15^ These recent results established a new paradigm for curved polyaromatic ligands in alkali metal binding and opened new opportunities for design and synthesis of novel mixed metal organometallic supramolecular products. However, the origin of the observed high negative shifts in ^7^Li NMR spectra was not understood and required additional attention.

Herein, we set to investigate the reduction reactions of corannulene using a binary alkali metal combination comprised of Li and Rb, the heavier congener of K. For the Li–Rb combination, we expect that a greater size mismatch of two alkali metals could not only facilitate the transformations observed in the Li–K–C_20_H_10_ system but also afford novel supramolecular products and open new reaction pathways that have not been seen before. We also expect that isolation and analysis of novel mixed metal organometallic products formed by the highly charged corannulene could help to shed light on the record negative shifts observed in ^7^Li NMR spectra. We therefore used a combination of NMR spectroscopy, X-ray crystallography and DFT theoretical methods for thorough investigation of these complex systems. As a result, we have been able to reveal a direct relation between the structures of supramolecular assemblies and coupling effects of the highly charged polyaromatic bowls at the molecular level and to correlate those with the observed ^7^Li NMR shifts.

## Results and discussion

Corannulene reacts with alkali metals, Li through Cs, to initially produce intense green solutions characteristic for the corannulene monoanion. The excess of alkali metal quickly reduces the resulting C_20_H_10_˙^–^ monoanion to the purple C_20_H_10_^2–^ dianion. In the case of Li metal, dianions can then be further reduced to tetraanions that form supramolecular [Li_5_(C_20_H_10_^4–^)_2_]^3–^ aggregates having characteristic ^7^Li NMR shifts for sandwiched Li^+^ ions (–11.70 ppm).^11,13^ While mono- and dianions of corannulene do not form such supramolecular species, they exhibit a variety of coordination modes that depend on the size of alkali metal ions and other experimental variables.^16,17^ The corannulene bowl is not flattened upon acquisition of the first electron but more pronounced changes can be seen upon addition of the second electron. In contrast, a significant bowl depth decrease and C–C bond length alteration pattern are observed for tetrareduced corannulene.^11,13,15,18^ These experimental observations are in line with multiple theoretical predictions.^11,19,20^

**Scheme 3:**
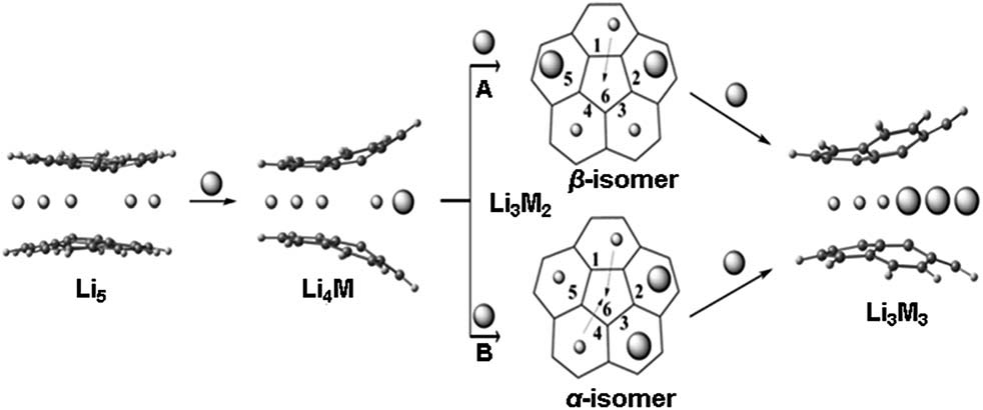
Representation of two pathways (A and B) for the Li insertion from the periphery (between 6-membered rings) into the inner space between two 5-membered rings of C_20_H_10_^4–^. Note that the pathways A and B have different energetic requirements. Li is shown as a sphere with small radius in contrast to a larger sphere that is used to represent larger alkali metals (M).

In Li_5_-sandwiches all five benzene rings of C_20_H_10_^4–^ are engaged in lithium ion binding leaving an internal space between two corannulene hub-sites empty. Notably, the distance between the centroids of 5-membered rings in the triple-decker Li_5_-products is 3.5 Å ^11^ and this may not be sufficient to accommodate an additional lithium ion in that space. For comparison, the separation between 5-membered rings in lithium cyclopentadienide, [(*η* ^5^-Cp)_2_Li]^–^, is *ca.* 4.0 Å,^21^ and the Li-Cp distances range from 3.8 to 4.1 Å in some other cyclopentadienide Li-organometallic compounds.^22,23^ We have recently demonstrated^15^ that in order to get access to the central cavity larger alkali metals, such as K, should be introduced into the reaction along with Li. The initially formed Li_5_-sandwich having both corannulene decks parallel to each other starts to open up upon stepwise substitution of small Li^+^ ions by larger K^+^ ions, leading to the products having angled corannulene decks (Scheme 3). This results in the opening of a channel which allows the insertion of Li^+^ ion from periphery into the previously inaccessible inner cavity of the sandwich (Scheme 3, pathways A and B). It is necessary to have two large alkali metal ions at the rim of the sandwich for the lithium insertion process to take place, as the first substitution step (formation of Li_4_K) does not provide a sufficient opening for such insertion. For the second step, there are two possible pathways, A and B (through the formation of α and β isomers), where rings 2 and 5 or 2 and 3 are occupied by larger alkali metals (Scheme 3). Our DFT calculations showed that in the Li–K systems the insertion occurs through the β-isomer only (pathway A).^15^

**Fig. 1:**
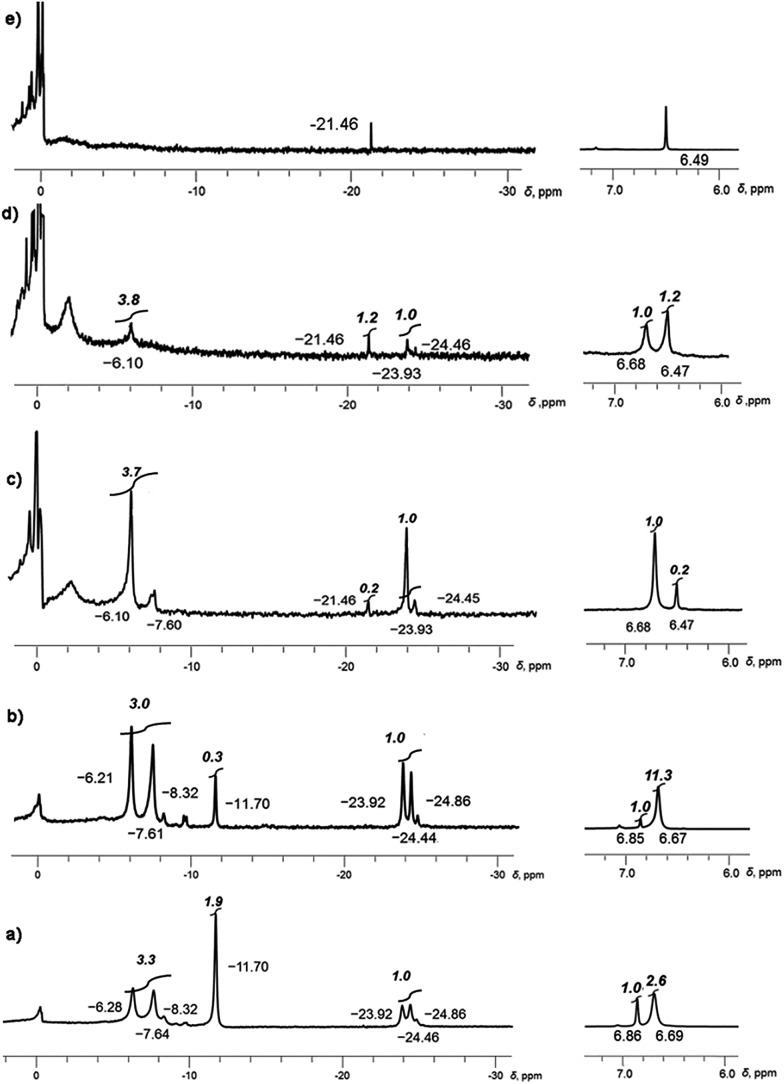
Monitoring of the dynamic progression from (a) to (e) of tetrareduced corannulene in the presence of Li and Rb metals in THF-*d*_8_ solution. Snapshots are taken with ^7^Li (left) and ^1^H (right) NMR at –80 °C.

Monitoring the reduction reaction of **1** with a mixture of Li and Rb metals in THF-*d*_8_ by NMR spectroscopy confirms the formation of supramolecular products formed by C_20_H_10_^4–^. The reaction seems to proceed faster than in the case of Li–K and substantial amounts of Li–Rb-sandwiches are quickly observed within a few hours (Fig. 1a).

**Fig. 2:**
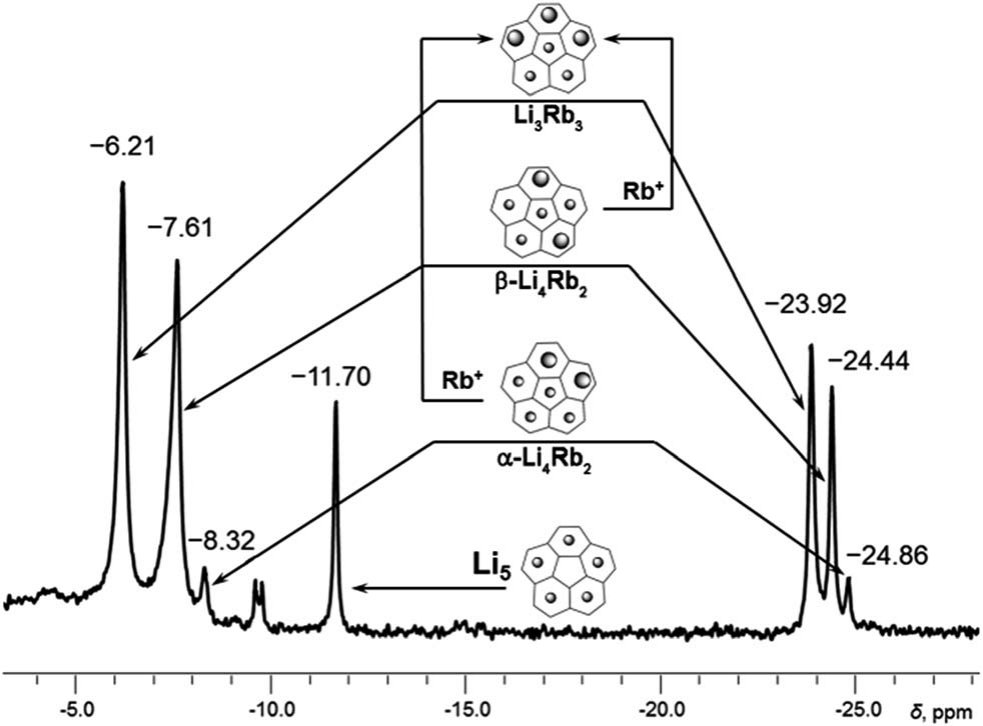
Subset of ^7^Li NMR spectrum from –4 to –28 ppm showing peaks of Li^+^ ions sandwiched between two C_20_H_10_^4–^ anions (for the full spectrum, see Fig. S2†). Relative peak integration: *ca.* 2.6 : 1 (–6.21 and –23.92 ppm), *ca.* 3.5 : 1 (–7.61 to –24.44 ppm) and *ca.* 3.0 : 1 (–8.32 to –24.86 ppm).

At the initial stage of reaction, which is determined by its characteristic brown-red color, two major broad peaks at –23.92 and –24.46 ppm and one very small peak at –24.86 ppm are observed in ^7^Li NMR spectrum (Fig. 1a). The significant upfield-shift of these peaks is associated with the Li^+^ ion squeezed between two 5-membered rings of tetrareduced corannulene, revealing the formation of several mixed metal Li–Rb sandwich-type assemblies. The latter peaks are assigned to the Li_3_Rb_3_ and Li_4_Rb_2_ products (α- and β-isomers, see Fig. 2 for schematic representation of the sandwich cores) based on integration of individual peaks and the possibility to crystallize these major products in the single-crystalline form, as reported below. It can be mentioned here that all NMR peaks coalesce into a single broad signal at temperatures ranging from +20 to –20 °C, showing the rapid lithium ion exchange between all molecular structures existing in solution (ESI, Fig. S1†).

The ^7^Li signal at –11.70 ppm in Fig. 1a belongs to the monometallic Li_5_-product in which five Li^+^ ions are occupying the sites in between the benzene rings of C_20_H_10_^4–^ (Scheme 2a). Additional peaks between –6.2 and –8.3 ppm are the signals of lithium ions located between the same 6-membered ring sites but belonging to the mixed metal Li–Rb sandwiches (similar to the reported Li–K aggregates).^15^

In contrast, the ^7^Li NMR signals of Li^+^ ions coordinating to the exterior of triple-decker sandwiches (filling the open concave cavities of C_20_H_10_^4–^ bowls) appear as broad peaks in the range between –4 to –6 ppm. The small intensity of these peaks observed in this work suggests that the larger Rb^+^ ions are preferred over smaller Li^+^ ions as the external charge-compensating cations. The ^7^Li NMR peaks around 0 ppm belong to the solvent-separated Li(THF)_*x*_^+^ species. No other peaks are observed in the +20 to –30 ppm range (Fig. S2†). Notably, the ratio of [Li_5_(C_20_H_10_^4–^)_2_]^3–^ to the mixture of all Li–Rb sandwiches is 0.38 to 1. This is consistent with the ^1^H NMR spectra which also show a 0.38 ratio of the representative peaks at 6.86 ppm (the signal of C_20_H_10_^4–^ in Li_5_) and 6.69 ppm (a broader peak associated with all mixed Li–Rb products). It should be mentioned here that the precise time assignment to a particular point in the series of dynamic NMR measurements is problematic due to very strong dependence of the system reactivity on very minor and hard-to-control experimental variations. For example, we observed that diminutive changes in purity of commercial metals (from the same supplier) play a drastic role in the reaction development. The NMR spectra represented in Fig. 1 show the reaction progression in the time frame of *ca.* 30 hours. These processes may be much slower (up to 3–5 days), if the quality of reagents is just slightly less perfect. In some cases, the reaction could be fully decomposed at an early stage not allowing for the final product to be obtained, indicating poor quality of the reagents used.

The next measurement (Fig. 1b) shows a smaller amount of the initially formed Li_5_-product and subsequent growth of the Li–Rb sandwiches, as illustrated by a better defined shape of the corresponding NMR signals. Therefore, a more detailed discussion of this spectrum is carried out below based on Fig. 2. It should be noted that the precise peak integration is still slightly hindered by peak overlaps and the elevated background downfield from *ca.* –10 ppm. The signal of external lithium ions of the Li_5_-sandwich, appearing as a very broad peak between –4 to –6 ppm, and the corresponding signals of external Li^+^ ions bound to other sandwich products existing in the mixed metal system all contribute to the elevated background.

Since the ^7^Li signal at –11.70 ppm belongs to the Li_5_-sandwich, containing lithium ions only,^11^ the close small peak at –9.62 ppm is assigned to the product resulting from the first substitution of one sandwiched Li^+^ ion by a Rb^+^ ion (to form a Li_4_Rb core). No peak correlates with this one in the region of –20 to –30 ppm in the ^7^Li NMR spectrum and, as expected, there is no internal lithium insertion at this stage. This transient species undergoes further substitution and thus does not accumulate in any significant amount in solution (Scheme 3).

The second substitution of Li by Rb leads to two Li_3_Rb_2_ isomers that open a path for the internal lithium insertion (pathways A and B, Scheme 3). After insertion, the resulting α- and β-isomers of Li_3_Rb_2_ have an open coordination site between the two benzene rings of corannulene decks and can be abbreviated for clarity as α-Li_3_Rb_2_□ and β-Li_3_Rb_2_□. Subsequent addition of Li^+^ to this open site provides α-Li_4_Rb_2_ and β-Li_4_Rb_2_ isomers, respectively. Final substitution of this loose Li^+^ ion by Rb^+^ affords the Li_3_Rb_3_-product (Fig. 2). Since the Li_3_Rb_3_ sandwich is a single product of both insertion pathways, the corresponding ^7^Li NMR signal at –23.92 ppm is notably growing with time (Fig. 1a–c). Three peaks at –6.21, –7.61, and –8.32 ppm can be identified in the ^7^Li NMR spectrum with a related triad of peaks having the same relative intensities being observed at the highly-negative region at –23.92, –24.44, and –24.86 ppm. Notably, the signals from these two triads correlate really well and therefore should belong to the same type of supramolecular sandwiches, as assigned in Fig. 2 with arrows. The triad on the right is associated with the Li^+^ ion internally located between the central 5-membered rings of C_20_H_10_^4–^, while the signals on the left stem from the Li^+^ ions sandwiched between the peripheral 6-membered rings of tetrareduced corannulene. The relative integration of peaks is *ca.* 2.6, 3.5, and 3.0 to 1 in pairs of –6.21/–23.92, –7.61/–24.44, and –8.32/–24.86 ppm, respectively. Slight overestimation of the integrated intensity may suggest that Li^+^ ions located at the sandwich periphery exchange with external environment. A small shoulder of the peak at –7.61 ppm may be indicative of differentiation between two peripheral Li^+^ ion sites observed in β-Li_4_Rb_2_.

It should be mentioned here that the insertion mechanism through the α-isomer of Li_3_Rb_2_ (pathway B, Scheme 3) is much less energetically favourable. The internal lithium migration for β-isomer was found to be barrierless, whereas for α-isomer the barrier was calculated to be +15.48 kcal mol^–1^.^24^ As a result, the corresponding NMR signals of α-isomer are always much less intense, being at about 10–20% relative to the β-isomer. In the case of Li–K-aggregates, the formation of α-isomer of the Li_3_K_2_ composition was not observed at all, thus showing that a larger Rb^+^ ion opens a new reaction pathway in comparison with K^+^.

After the Li_5_-sandwich is fully consumed in the stepwise substitution reactions (as seen by the disappearance of the 6.85 ppm peak in ^1^H and –11.70 ppm peak in ^7^Li NMR spectra, Fig. 1c) the major product in solution is the Li_3_Rb_3_ sandwich, with the β-isomer of Li_4_Rb_2_ being a minor component of the mixture. Interestingly, there is a new peak appearing at –21.46 ppm which is assigned to the fully substituted product having five rubidium ions at the rim (LiRb_5_). Similar to the Li–K system (^7^Li NMR signal of LiK_5_ appears at *ca.* –22.40 ppm), this is the only mixed metal aggregate that can be observed at elevated temperatures (already clearly visible at *ca.* 0 °C, Fig. S4†) due to negligible exchange of the central Li^+^ ion trapped inside the sandwich.

In the corresponding ^1^H NMR spectra, the protons of C_20_H_10_^4–^ in Li_3_Rb_3_ and Li_4_Rb_2_ aggregates are observed at 6.68 ppm, while a sharp peak, corresponding to the LiRb_5_ sandwich, appears at 6.47 ppm. As concluded from the relative peak integration, LiRb_5_ constitutes approximately 20% of the reaction mixture in solution. At this point, the amount of solvent-separated Li(THF)_*x*_^+^ species (broad peak at *ca.* 0 ppm) and non-sandwiched lithium ions bound to the external surface of C_20_H_10_^4–^ (very broad, 0 to –4 ppm) is quite significant. This is also accompanied by the appearance of some precipitation–decomposition products in the NMR ampoule.

Notably, NMR monitoring shows that the α-isomer of Li_4_Rb_2_ can no longer be seen and the amount of the corresponding β-isomer is very small. At the final stage, LiRb_5_ is revealed as the major component in the mixture (Fig. 1d), and it finally becomes the only product observed in solution (Fig. 1e). The formation of LiRb_5_ is accompanied by the appearance of a single peak at –21.46 ppm in ^7^Li NMR and at 6.49 ppm in ^1^H NMR spectra. It should be mentioned here that a substantial amount of unidentified precipitate is observed in the NMR ampoule at this stage.

### X-ray crystallographic studies

**Fig. 3:**
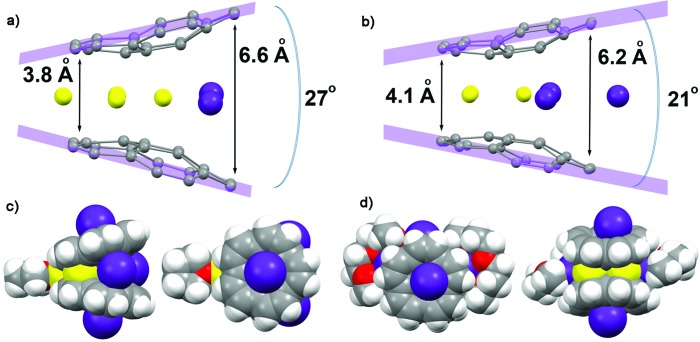
Molecular structures of (a) β-Li_3_Rb_2_□–Li and (b) Li_3_Rb_3_ products showing the sandwich opening along with the shortest and longest distances between the corannulene decks. Two orthogonal space filling views of the respective molecular structures (c) and (d), showing all eight alkali metal ions inside and outside of the sandwich and the immediate coordination environment of these molecules.

As revealed by NMR studies (Fig. 1a–c), the mixed metal Li–Rb sandwiches are represented by two major contributors existing in solution, namely Li_3_Rb_3_ and β-Li_4_Rb_2_. From these systems, by applying suitable crystallization techniques (see ESI†), we were able to crystallize two products, [{Rb(THF)_2_}_2_]//[Li_3_Rb_2_□(C_20_H_10_)_2_{Li(THF)}] (**2**) and [{Rb(diglyme)}_2_]//[Li_3_Rb_3_(C_20_H_10_)_2_(diglyme)_2_]·0.5THF (**3**·0.5THF). Importantly, while the Li_3_Rb_3_ sandwich constitutes a close analogue of the previously reported Li_3_K_3_ product,^15^ the Li_3_Rb_2_-sandwich having an empty coordination site between corannulene decks is observed for the first time. To clearly indicate this open site (abbreviated as □) and the β-disposition of two adjacent rubidium ions we designate this novel sandwich as β-Li_3_Rb_2_□. The single crystal X-ray diffraction analyses revealed that geometric parameters of C_20_H_10_^4–^ anions in the Li_3_Rb_3_ and β-Li_3_Rb_2_ products (Fig. 3) are similar to those in the Li_3_K_3_ sandwich (see Table S2†).

It should be emphasized here that the fact that the transient β-Li_3_Rb_2_□ sandwich can be isolated (Fig. 3a and c) provides compelling experimental evidence for the proposed Li-insertion mechanism. The sandwich opening (measured as an angle between the mean planes passing through the rim of two π-bowls) in the case of β-Li_3_Rb_2_□ is greater than in the Li_3_Rb_3_ or Li_3_K_3_ sandwiches (27° *vs.* 21° and 20°, respectively). The distance between the rim C-atoms at the sandwich opening side is about 6.6 Å, which is substantially longer than the corresponding separations in Li_3_Rb_3_ (Fig. 3b) or Li_3_K_3_ cases (6.2 and 6.1 Å, respectively).

In β-Li_3_Rb_2_□, the fourth Li^+^ ion (also having a coordinated THF molecule) is closely approaching the sandwich (to show this side binding, the sandwich is abbreviated as β-Li_3_Rb_2_□–Li) from the Li_3_-triangular side (Fig. 3c), thus forming a Li_4_ parallelogram with the Li···Li contacts of 2.86 Å inside the sandwich and of 2.92 Å outside the sandwich. This additional lithium cation brings both corannulene bowls to a very short distance of 3.8 Å (compared with 4.1 Å in Li_3_Rb_3_). In Li–Rb sandwiches in **2** and **3**, both external concave cavities of C_20_H_10_^4–^ are filled with rubidium ions that participate in the formation of a 1D network through the shared external solvent molecules (Fig. S5†). In **2**, these 1D chains are further packed into 2D sheets *via* additional C–H···π interactions (Fig. S6†).

**Fig. 4:**
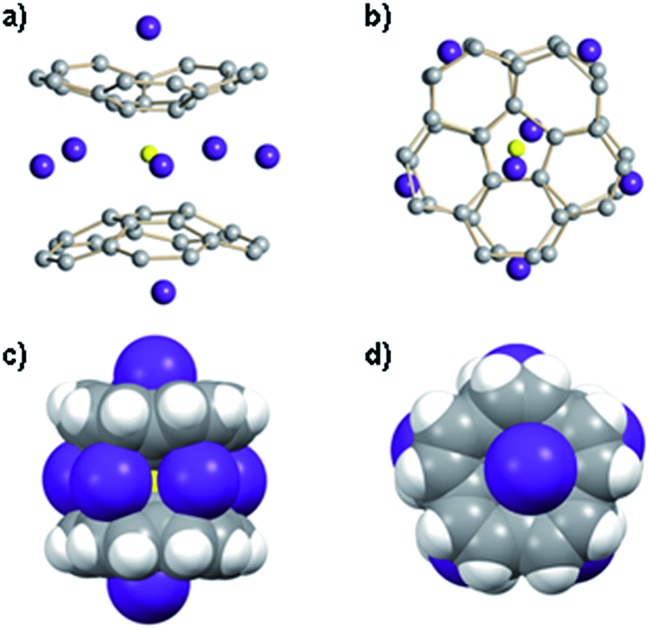
Orthogonal views of the LiRb_5_-core structure with two Rb^+^ ions occupying the outside concave cavities of tetrareduced corannulene bowls shown with ball-and-stick (a) and (b) and with space filling models (c) and (d).

Crystallization of the final product in this series of dynamic transformations, namely of LiRb_5_, has been very problematic. By the time this sandwich appears as the only product in solution, which is determined by its characteristic red color, there is a significant amount of precipitation–decomposition observed in the system. Removal of this unidentified solid affords rather dilute solutions, which do not allow efficient crystal growth of LiRb_5_. After numerous attempts we were able to isolate the single crystals of LiRb_5_ sandwich, but even the best crystals show diffraction only up to *ca.* 1 Å resolution. Nevertheless, these X-ray diffraction data allowed us to confirm the LiRb_5_ core structure of this product (Fig. 4), as predicted based on the stepwise alkali metal substitution mechanism and NMR data.

### ^7^Li NMR shifts and structural correlations

The record negative ^7^Li NMR shifts characteristic of supramolecular Li–K and Li–Rb sandwich products formed by C_20_H_10_^4–^ anions are worthy of special discussion. Such unprecedented shifts of the central Li^+^ ion occupying the cavity between the two hub-sites of corannulene tetraanions (signals range from –21 ppm to –25 ppm) are due to strong shielding of this internal cation from the external magnetic field. This implies that the centrally located Li^+^ ion is surrounded by some electron density, which clearly separates and distinguishes it from other alkali metal ions (Li, K, or Rb) sandwiched in-between the peripheral 6-membered rings of C_20_H_10_^4–^ anions. Importantly, this provides the first indication that some electronic communication should exist between the negatively charged corannulene decks separated by an alkali metal layer in the triple-decker organometallic products. The most plausible way for such electronic exchange should be through the orbital interactions between the two tetrareduced π-bowls. This is clearly confirmed by the observation that the shift of ^7^Li NMR signal of the central Li^+^ cation correlates with the distance between two corannulene decks in the isolated sandwich structures.

In Li_3_K_3_, the central Li^+^ ion resonates at –24.48 ppm but the signal is shifted to *ca.* –22.40 ppm in LiK_5_ (the separations between the hub-sites of two C_20_H_10_^4–^ decks are 3.8 and 4.0 Å, respectively).^15^ The centrally located Li^+^ ions in Li_3_Rb_3_ and LiRb_5_ products appear to be deshielded (–23.92 and –21.46 ppm) in comparison with their Li_3_K_3_ and LiK_5_ analogues, which is consistent with the size of Rb^+^ ions requiring more space than K^+^ ions. As a result, the corresponding separations between two corannulene bowls in Li_3_Rb_3_ and LiRb_5_ are 4.1 and 4.2 Å, respectively. Clearly, Rb^+^ ions move the charged decks farther apart and thus weaken communication between two π-bowls in the triple-decker aggregates. Consequently, the central Li^+^ cation becomes less shielded and the corresponding ^7^Li NMR signals shift downfield for sandwich structures having higher amount of heavier alkali metals.

The close analogy can be drawn with the strong shielding effect of the C_60_-fullerene hexaanion. The ^3^He atom inside the C_60_^6–^ cage was found to be significantly more strongly shielded (by *ca.* 20 ppm) than any other previously reported encapsulated ^3^He atom, suggesting the ability of electrons to move freely about the surface of a charged spheroidal π-system.^25^ In the case of mixed alkali metal sandwich-type assemblies formed by highly charged corannulene, electrons can also move freely about the π-bowl surface of **1** ^4–^, but in addition to that, they are able to move from one surface to the other, as we found here for the first time.

### DFT computational studies

To get further theoretical insights into the experimentally observed electronic communication between the highly charged corannulene anions, we carried out DFT calculations at the PBE0/def2-TZVPP(Li, Rb)//cc-pVDZ(C, H) level of theory. The choice of the model system was discussed elsewhere.^15^

**Fig. 5:**
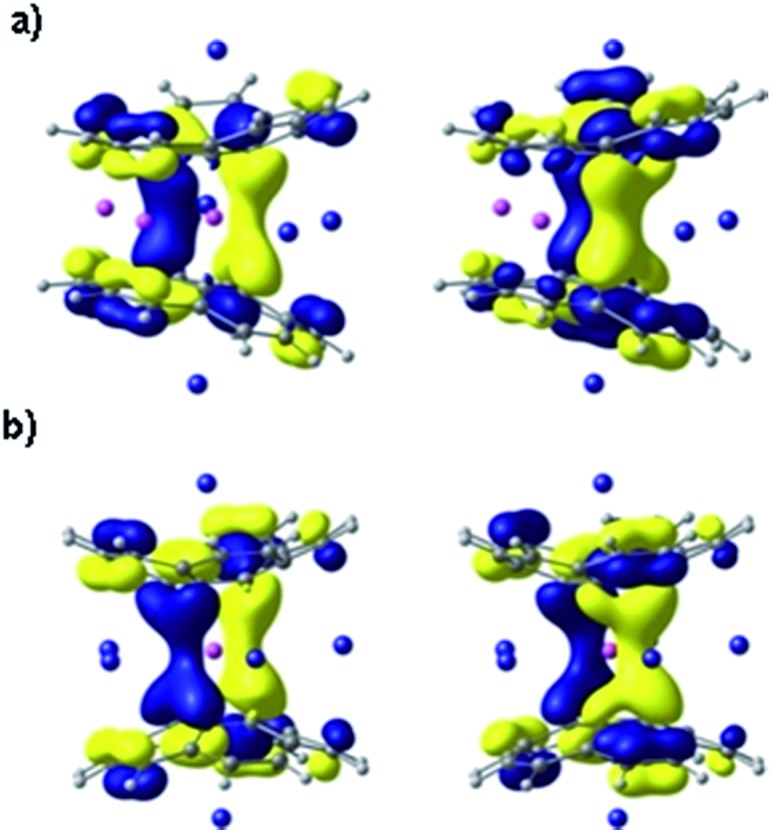
Canonical MOs, HOMO-10 (left) and HOMO-11 (right) in Li_3_Rb_3_ (a) and LiRb_5_ (b) systems, showing coupling and sharing electron density between two tetrareduced corannulene bowls. MOs are presented with an isosurface of 0.03 a.u.

Indeed, theoretical calculations revealed^24^ that π-systems of two tetrareduced corannulene bowls are coupled with each other, forming shared electronic density that is mainly concentrated in the region between two 5-membered rings. The central lithium cation is thus wrapped into a negatively charged cocoon, which seems responsible for a record high shielding effect observed in ^7^Li NMR. The corresponding orthogonal molecular orbitals representing delocalization of electronic density between the C_20_H_10_^4–^ bowls are shown in Fig. 5.

Theoretical analysis of components of shielding tensor revealed that the main difference between ^7^Li NMR signals of the lithium ions placed in-between six-membered rings and of the central Li^+^ ion comes from the paramagnetic term of the Ramsey formula (*σ* ^tot^ = *σ* ^d^ + *σ* ^p^). The paramagnetic term involves mixing of ground and excited states of the molecule due to the magnetic field, and it is rather sensitive to the molecular electronic structure. Thus, the main reason for such a large shielding of the central Li^+^ ion, in comparison with those located between six-membered rings of C_20_H_10_^4–^, should arrive from the electronic structure of the sandwich and, more precisely, from the local electronic environment of the cation. MOs presented in Fig. 5 are found to provide the largest contributions to the *σ* ^p^ component of the shielding tensor. These MOs clearly show extended delocalization around the central Li^+^ ion and no delocalization for the lithium(s) sitting between peripheral 6-membered rings. At the same time, the calculated charges of lithium cations of both types indicate essentially no charge transfer from the highly negatively charged corannulene tetraanions to Li^+^ ions. The NBO charges are very similar (+0.88 and +0.91, respectively, in Li_3_Rb_3_ and +0.87 in LiRb_5_). The larger distance between two bowls makes the coupling between them weaker, as suggested from experimental data. Consequently, this results in a downfield-shift of the ^7^Li NMR signal from the central Li^+^ ion. The corresponding component of the shielding tensor was found to show the trend that is opposite to the distance between two C_20_H_10_^4–^ anions. Thus, the signal from the central lithium cation could be used for qualitative evaluation of the strength of coupling between the charged bowls in sandwich-like aggregates.

## Conclusions

Several remarkable triple-decker organometallic aggregates having a mixed metal core (Li_3_Rb_2_□, Li_3_Rb_3_, and LiRb_5_) sandwiched between two tetrareduced corannulene decks have been isolated in this work, following the NMR studies of their dynamic transformations in solutions. These sandwiches have the central cavity located in between of the hub-sites of two C_20_H_10_^4–^ decks occupied by an internal Li^+^ ion that exhibits the record high negative shift (up to –25 ppm) in ^7^Li NMR spectra. Theoretical investigation of these unique systems revealed that coupling of two highly-charged corannulene bowls results in a shared region of high negative electron density around the central lithium ion that is responsible for a record shielding effect observed in ^7^Li NMR spectra. Analysis of three new sandwich structures allowed us to identify the trend: the larger separation between two π-bowls makes their coupling to be weaker, which is accompanied by the down-field shift of the corresponding ^7^Li NMR signal from the internally encapsulated Li^+^ ion. Consequently, these ^7^Li NMR signals can be used for qualitative evaluation of the strength of coupling between the charged carbon bowls in sandwich-like supramolecular aggregates. The higher the negative chemical shift is the stronger coupling should be expected.

## References

[cit1] Imahori H. and Umeyama T., Applications of Supramolecular Ensembles with Fullerenes and CNTs: Solar Cells and Transistors, in Supramolecular Chemistry of Fullerenes and Carbon Nanotubes, ed. N. Martin and J.-F. Nierengarten, Wiley-VCH Verlag GmbH & Co. KGaA, 2012, p. 390.

[cit2] Lee S. W., Yabuuchi N., Naoaki G., Gallant B. M., Chen S., Shuo K., Byeong-Su H., Hammond P. T., Yang S.-H. (2010). Nat. Nanotechnol..

[cit3] Xiang X.-D., Hou J. G., Crespi V. H., Zettl A., Cohen M. L. (1993). Nature.

[cit4] (c) Fragments of Fullerenes and Carbon Nanotubes: Designed Synthesis, Unusual Reactions, and Coordination Chemistry, ed. M. A. Petrukhina and L. T. Scott, John Wiley & Sons, New Jersey, 2012, p. 413.

[cit5] Colson J. W., Woll A. R., Mukherjee A., Levendorf M. P., Spitler E. L., Shields V. B., Spencer M. G., Park J., Dichtel W. R. (2011). Science.

[cit6] Yamago S., Watanabe Y., Iwamoto T. (2010). Angew. Chem., Int. Ed..

[cit7] Filatov A. S., Petrukhina M. A. (2010). Coord. Chem. Rev..

[cit8] (g) Aprahamian I. and Rabinovitz M., The Lithium Metal Reduction of π-Conjugated Hydrocarbons and Fullerenes, in The Chemistry of Organolithium Compounds, ed. Z. Rappoport and I. Marek, John Wiley & Sons, UK, 2006, p. 477.

[cit9] Baumgarten M., Gherghel J. L., Wagner M., Weitz A., Rabinovitz M., Cheng P.-C., Scott L. T. (1995). J. Am. Chem. Soc..

[cit10] Ayalon A., Rabinovitz M., Cheng P.-C., Scott L. T. (1991). Angew. Chem., Int. Ed. Engl..

[cit11] Zabula A. V., Filatov A. S., Spisak S. N., Rogachev A. Yu., Petrukhina M. A. (2011). Science.

[cit12] Bruno C., Benassi R., Passalacqua A., Paolucci F., Fontanesi C., Marcaccio M., Jackson E. A., Scott L. T. (2009). J. Phys. Chem. B.

[cit13] Zabula A. V., Spisak S. N., Filatov A. S., Petrukhina M. A. (2012). Organometallics.

[cit14] Gerald R. E., Klingler R. J., Sandi G., Johnson C. S., Scanlon L. G., Rathke J. W. (2000). J. Power Sources.

[cit15] Filatov A. S., Zabula A. V., Spisak S. N., Rogachev A. Yu., Petrukhina M. A. (2014). Angew. Chem., Int. Ed..

[cit16] Filatov A. S., Sumner N. J., Spisak S. N., Zabula A. V., Rogachev A. Y., Petrukhina M. A. (2012). Chem. - Eur. J..

[cit17] Spisak S. N., Zabula A. V., Ferguson M. V., Filatov A. S., Petrukhina M. A. (2013). Organometallics.

[cit18] Zabula A. V., Spisak S. N., Filatov A. S., Petrukhina M. A. (2012). Angew. Chem., Int. Ed..

[cit19] Spisak S. N., Zabula A. V., Filatov A. S., Rogachev A. Yu., Petrukhina M. A. (2011). Angew. Chem., Int. Ed..

[cit20] Green J. R., Dunbar R. C. (2011). J. Phys. Chem. A.

[cit21] Harder S., Prosenc M. H. (1994). Angew. Chem., Int. Ed. Engl..

[cit22] Stalke D. (1994). Angew. Chem., Int. Ed. Engl..

[cit23] Foy J. T., Wilkes E. B., Aprahamian I. (2012). CrystEngComm.

[cit24] Energetic evaluation was performed with the help of *x*DH-PBE0 functional for PBE0-optimized geometries (see ESI for more details). Correlation-consistent full-electron basis sets of double-*ζ* quality (cc-pVDZ) were applied for description of light elements (C and H), while def2-TZVP basis sets were applied for all metal atoms (combined with effective core potential for Rb). Importantly, for the α-isomer two different migration processes were observed. Here, we consider only one having the lowest barrier (Scheme 3)

[cit25] Shabtai E., Weitz A., Haddon R. C., Hoffman R. E., Rabinovitz M., Khong A., Cross J. R., Saunders M., Cheng P.-C., Scott L. T. (1998). J. Am. Chem. Soc..

