# Supplementary material for: Self-assembly of tetrareduced corannulene with mixed Li–Rb clusters: dynamic transformations, unique structures and record 7Li NMR shifts
Source: Chem Sci. 2014 Dec 16;6(3):1959–66. doi: 10.1039/c4sc03485f (PMC5496504; doi:10.1039/c4sc03485f)
Supplement: Supplementary file 1 [file SC-006-C4SC03485F-s001.pdf]

Electronic Supplementary Material (ESI) for Chemical Science.
This journal is © The Royal Society of Chemistry 2014

Supporting Information

Self-Assembly of Tetrareduced Corannulene with Mixed Li/Rb Clusters: Dynamic Transformations, Unique Structures and Record ^{7}Li NMR Shifts

Alexander S. Filatov,[a] Sarah N. Spisak,[a] Alexander V. Zabula,[b]

James McNeely,[c] Andrey Yu. Rogachev,[c] Marina A. Petrukhina[a,*]

[a] Department of Chemistry, University at Albany, State University of New York, Albany, NY 12222, USA

[b] Department of Chemistry, University of Wisconsin, Madison, WI 53706, USA

[c] Department of Biological and Chemical Sciences, Illinois Institute of Technology, Chicago, IL 60616, USA

I. Experimental Part S3
Materials and Methods S3
Preparation of [{Rb(THF)$_2$}$_2$]//[Li$_3$Rb$_2$(C$_{20}$H$_{10}$)$_2${Li$^+$(THF)}] (2) S3
Preparation of [{Rb(diglyme)}$_2$]//[Li$_3$Rb$_3$(C$_{20}$H$_{10}$)$_2$)(diglyme)$_2$] · 0.5THF (3 · 0.5THF) S4
Figure S1. Coalescence of all ^{7}Li NMR peaks at temperatures ranging from +20 to −20 ºC S4
Figure S2. ^{7}Li NMR spectrum shown from +20 to −30 ppm at −80 ˚C S4
Figure S3. ^{1}H NMR (left) and ^{7}Li NMR (right) spectra for the *in situ* generated $C_{20}H_{10}^{4-}$ with Li/Rb in THF-d_8 after 1 day S5
Figure S4. Variable-temperature ^{7}Li NMR spectra of LiRb$_5$-sandwich S5
II. Crystal Structure Solution and Refinement S6
Figure S5. 1D chains formed through the external solvent molecules and Rb ions filling the concave cavities of the $C_{20}H_{10}^{4-}$ bowls in (a) Li$_4$Rb$_2$ and (b) Li$_3$Rb$_3$ products S6
Figure S6. 2D sheets in Li$_4$Rb$_2$ formed through additional C-H···π interactions of external THF molecules and charged π-bowls of neighboring 1D chains S7
Table S1. Crystal and refinement data of **2** and **3** S7
Table S2. Selected bond lengths of Li$_5$, **2**, and **3** (in Å) S8
III. Calculations S9
Table S3. Cartesian coordinates for Li$_3$Rb$_3$ optimized at the PBE0/def2-TZVP(Rb,Li)//cc-pVDZ(C,H) level of theory S10
Table S4. Cartesian coordinates for LiRb$_5$ optimized at the PBE0/def2-TZVP(Rb,Li)//cc-pVDZ(C,H) level of theory S11
Table S5. Absolute energies (in Hartree) for Li$_3$Rb$_3$ and LiRb$_5$ products calculated at the PBE0/def2-TZVP(Rb,Li)//cc-pVDZ(C,H) level of theory S13
Figure S7. Schematic representation of α- and β-isomers of Li$_3$Rb$_2$ along with possible migration pathways S13
Figure S8. Schematic representation of internal lithium migration for Li$_3$Rb$_2$ system S14

Table S6. Cartesian coordinates for the parent α-Li_3Rb_2 sandwich optimized at the PBE0/def2-TZVP(Rb,Li)//cc-pVDZ(C,H) level of theory S14
Table S7. Cartesian coordinates for the transition state in α-Li_3Rb_2 optimized at the PBE0/def2-TZVP(Rb,Li)//cc-pVDZ(C,H) level of theory S16
Table S8. Cartesian coordinates for the product of migration in α-Li_3Rb_2 optimized at the PBE0/def2-TZVP(Rb,Li)//cc-pVDZ(C,H) level of theory S17
Table S9. Absolute energies (in Hartree) for parent compound, transition state and product of insertion for α-Li_3Rb_2 calculated at different levels of theory S19
Table S10. Cartesian coordinates for the product of migration in β-Li_3Rb_2 optimized at the PBE0/def2-TZVP(Rb,Li)//cc-pVDZ(C,H) level of theory S19
Table S11. Absolute energies (in Hartree) for the product of insertion for β-Li_3Rb_2 calculated at different levels of theory S21
IV. References S21

I. Experimental Part

Materials and Methods. All manipulations were carried out using break-and-seal[1] and glove-box techniques under an atmosphere of argon. Solvents (THF and hexanes) were dried over Na/benzophenone and distilled prior to use. Diglyme and THF-d_8 were dried over NaK_2 alloy and vacuum-transferred. Alkali metals (Li and Rb) were purchased from Strem Chemicals. Corannulene was prepared as described previously[2] and sublimed at 175 °C prior to use. The ^{1}H NMR spectra were measured on a Bruker AC-400 spectrometer at 400 MHz and were referenced to the resonances of the corresponding solvent used. The ^{7}Li NMR spectra were measured at 155.5 MHz (LiCl in THF-d_8). All shifts are reported in ppm. Elemental analyses were performed by Complete Analysis Laboratories, Inc., Parsippany, NJ. In order to prepare NMR samples, THF-d_8 (0.7 mL) was added to a NMR probe containing excess Li (approx. 10 eq.), excess Rb (approx. 10 eq.), and corannulene (10 mg, 0.04 mmol). The ampule was then sealed. Once the formation of the tetrareduced corannulene was observed, which is indicative by the brown color, NMR measurements were conducted starting from room temperature and going down to −80 ˚C.

Preparation of [{Rb(THF)$_2$}$_2$]//[$Li_3Rb_2(C_{20}H_{10})_2${Li^+(THF)}] (2)

THF (3 mL) was added to a flask containing excess Li, excess Rb, and corannulene (15 mg, 0.06 mmol). The deep green solution was stirred at room temperature for 8 hours resulting in a deep brown mixture. The brown mixture was filtered, layered with hexanes (4 mL), and kept at 10 °C. Crystals (blocks) of **2** were collected in 50 hours. The brown solution was decanted, and the crystals were washed several times with hexanes and dried *in vacuo*. Yield: 50%. Anal. Calcd for $C_{60}H_{60}Li_4O_5Rb_4$: C; 58.55, H; 4.91; Found: C; 58.32; H; 5.05; ^{1}H NMR (400 MHz, THF-d_8, −80 °C, ppm): δ = 6.68 (broad peak). ^{7}Li NMR (155.5 MHz, THF-d_8, −80 °C, ppm): δ = −24.48.

Preparation of [{Rb(diglyme)}$_2$]//[$Li_3Rb_3(C_{20}H_{10})_2$)(diglyme)$_2$] (3)

Diglyme (3.0 mL) was added to a flask containing excess Li, excess Rb, and corannulene (15 mg, 0.06 mmol). The deep green solution was stirred at room temperature for 26 hours resulting in a deep red-brown mixture. The red-brown mixture was filtered, layered with THF:hexanes (1:10, 4 mL), and kept at 10 °C. Crystals (plates) of **3** · 0.5THF were collected in 36 hours. The red-brown solution was decanted, and the crystals were washed several times with hexanes and dried *in vacuo*. Yield: 60%. ^{1}H NMR (400 MHz, THF-d_8, −80 °C, ppm): δ = 6.68 (broad peak). ^{7}Li NMR (155.5 MHz, THF-d_8, −80 °C, ppm): δ = −23.93. Anal. Calcd for $C_{64}H_{76}Li_3O_{12}Rb_5$: C; 51.75, H; 5.16; Found: C; 51.68; H; 5.09.

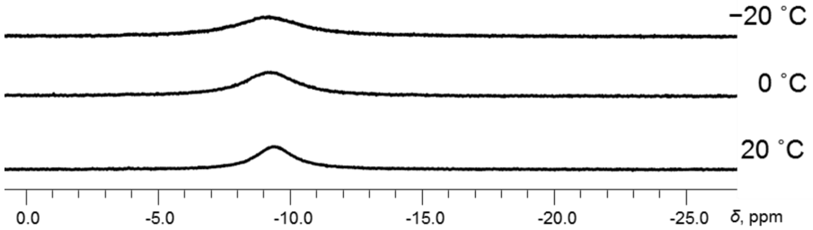

Figure S1. Coalescence of all ^{7}Li NMR peaks at temperatures ranging from +20 to −20 ºC.

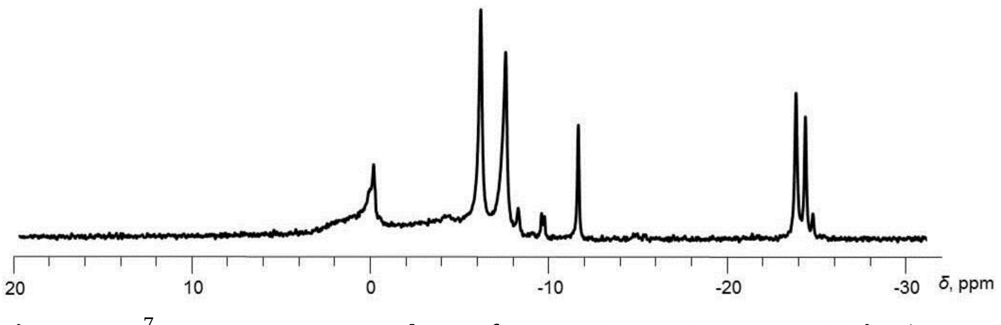

Figure S2. ^{7}Li NMR spectrum shown from +20 to −30 ppm at −80 ˚C (see Figure 2 in main text).

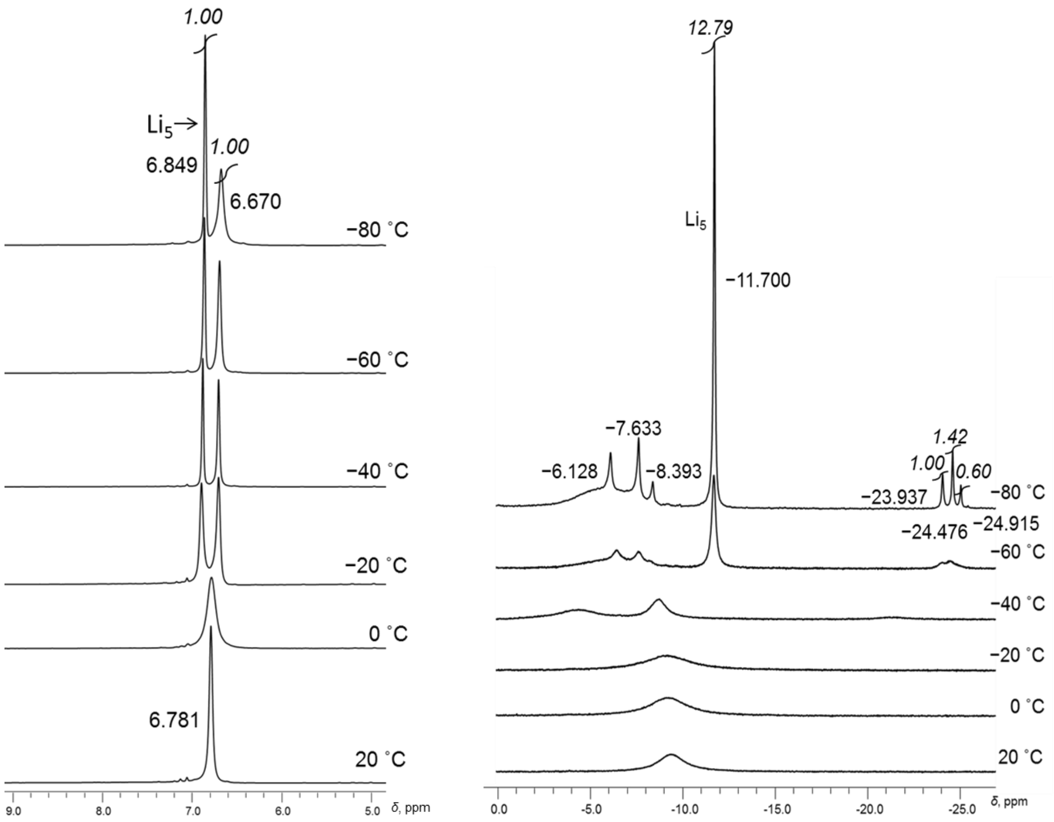

Figure S3. ^{1}H NMR (left) and ^{7}Li NMR (right) spectra for the *in situ* generated $C_{20}H_{10}^{4-}$ with Li/Rb in THF-d_8 after 1 day. These spectra were measured starting at room temperature and cooling to −80 ˚C.

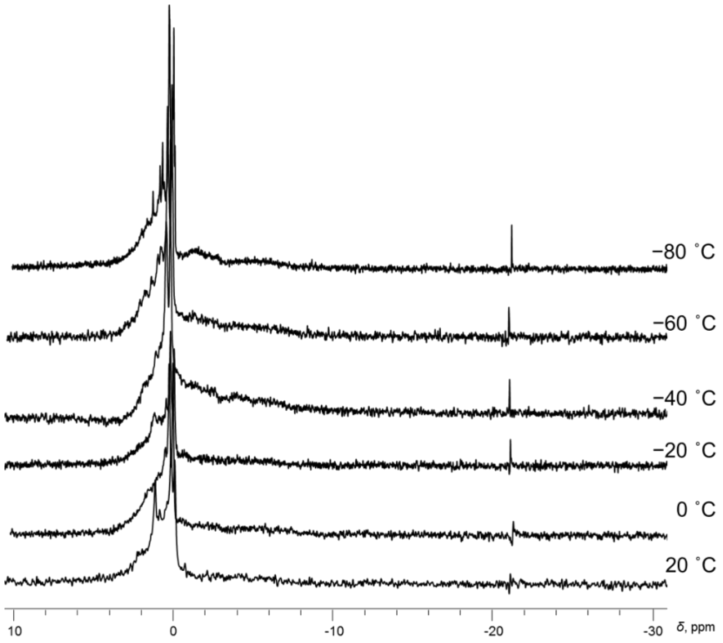

Figure S4. Variable-temperature ^{7}Li NMR spectra of LiRb$_5$-sandwich.

II. Crystal Structure Solution and Refinement

Data collections were performed on a Bruker SMART APEX CCD-based X-ray diffractometer with graphite-monochromated Mo-Kα radiation (λ = 0.71073 Å) at T = 100(2) K. Data reduction and integration were performed with the Bruker software package SAINT (version 8.27). Data were corrected for absorption effects using the empirical methods as implemented in SADABS. The structures were solved and refined by full-matrix least-squares procedures using the Bruker SHELXTL (version 6.14) software package. All atoms were refined with anisotropic thermal parameters. Hydrogen atoms were included in idealized positions for structure factor calculations. After numerous attempts we were able to isolate crystals of $LiRb_5$ sandwich but even for the best diffracting crystal the X-ray intensity data measured on a Bruker D8 VENTURE with PHOTON 100 CMOS detector system equipped with a Mo-target X-ray tube (λ = 0.71073 Å) were very weak and a full diffraction data set could only be collected to ca. 1 Å resolution. Nevertheless, these data revealed and confirmed the $LiRb_5$ core structure of the sandwich. Data: orthorhombic, $P2_12_12_1$, a = 14.531(3), b = 14.535(3), c = 33.157(7) Å, V = 7003(3) Å^3, T = 100(2) K. The corannulene core bowl depth is 0.97 Å and the Li-C(centroid of the 5-membered ring) = 2.10 Å.

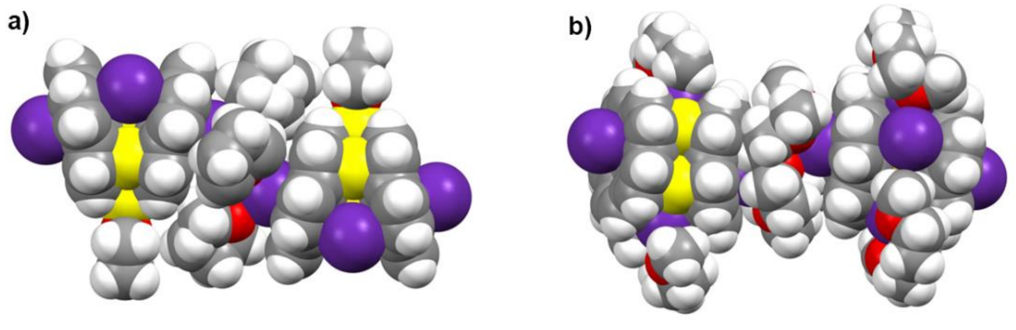

Figure S5. 1D chains formed through the external solvent molecules and Rb ions filling the concave cavities of the $C_{20}H_{10}^{4-}$ bowls in (a) Li_4Rb_2 and (b) Li_3Rb_3 products.

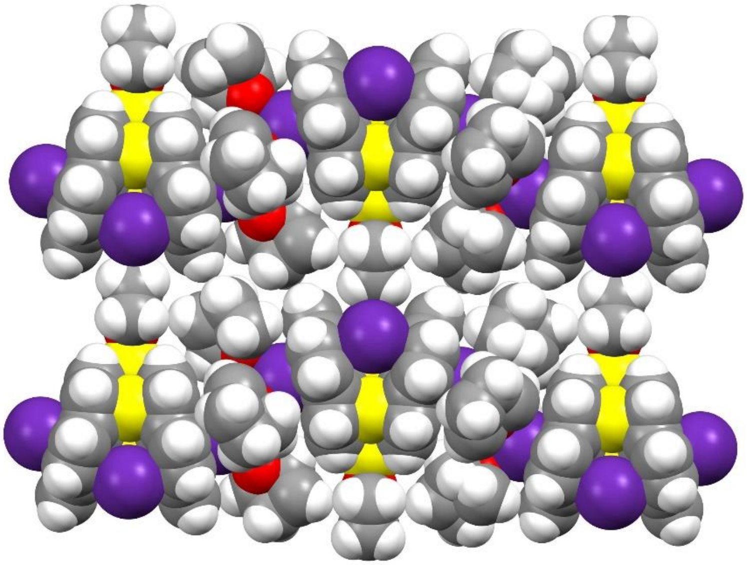

Figure S6. 2D sheets in the Li_4Rb_2 product formed through additional C-H···π interactions of external THF molecules and charged π-bowls of neighboring 1D chains (as shown in Fig. S5).

Table S1. Crystal and refinement data of **2** and **3**.

Parameter	**2**	**3**
Empirical formula	$C_{60}H_{60}Li_4O_5Rb_4$	$C_{66}H_{80}Li_3O_{12.5}Rb_5$
M_r	1230.72	1521.47
T (K)	100	100
Crystal system	Orthorhombic	Triclinic
Space group	*C* 2c 2	*P*-1
a (Å)	14.166(7)	10.064(3)
b (Å)	13.519(7)	15.270(4)
c (Å)	25.901(13)	22.129(6)
α (°)	90	90.416(3)
β (°)	90	93.102(3)
γ (°)	90	94.532(4)
V (Å^3)	4960(4)	3385.1(15)
Z	4	2
ρ_{calcd} [g·cm^{-3}]	1.648	1.493
μ [mm^{-1}]	3.975	3.647
2θ-range [deg]	3.14−50.04	3.24−50.70
data collected	17450	24628
R_{int}	0.1667	0.0815
Data/restraints/parameters	2347/142/207	12230/100/809

R_1, wR_2 indices [I>2σ(I)]	0.0846, 0.1934	0.0977,0.2150
R_1, wR_2 indices (all data)	0.1516, 0.2331	0.1703, 0.2399
Quality-of-fit	1.072	1.098
peak/hole [e·Å^{-3}]	2.624/-1.396	2.165/-0.917

$R_1 = \Sigma||F_o|-|F_c||/\Sigma|F_o|$; $wR_2 = [\Sigma[w(F_o^2-F_c^2)^2]/\Sigma[w(F_o^2)^2]]^{½}$;

Quality-of-fit = $[\Sigma[w(F_o^2-F_c^2)^2]/(N_{obs}-N_{params})]^{½}$.

Table S2. Selected bond lengths of Li_5, **2**, and **3** (in Å).

	**Li₅**	**2**	**3**
hub	1.390(3)–1.400(2)	1.382(12)−1.396(14)	1.386(17)−1.431(16)<br>1.380(17)−1.434(17)
spoke	1.421(2)–1.433(2)	1.442(19)−1.464(16)	1.412(17)−1.464(17)<br>1.415(17)−1.453(18)
rim	1.449(3)–1.490(2)	1.35(2)−1.431(18)	1.408(16)−1.453(18)<br>1.419(18)−1.466(19)
flank	1.429(3)–1.445(2)	1.42(2)−1.447(18)	1.394(19)−1.446(18)<br>1.405(19)−1.481(17)
$C_5\cdots C_5$**	3.517(2)	3.83(2)	3.820(17)
Li···C(η^5)	--	2.281(13)−2.287(13)	2.24(3)−2.33(3)
Li···C(η^6)	2.226(3)−2.718(4)	2.214(13)−2.732(13)	3.00(3)−3.52(3)
$Rb_{sand}\cdots C$	--	3.109(14)−3.735(14)	3.016(13)−3.590(13)
$Rb_{ext}\cdots C$	--	3.025(9)−3.624(9)	2.969(13)−3.397(13)
Li···Li	3.048(4)−3.100(4)	2.860(13)	3.00(3)−3.52(3)
Li···Rb	--	3.482(9)<br>4.093(9)	3.67(3)−4.02(3)
Rb···Rb	--	5.994(11)	4.233(13)<br>5.113(13)
bowl depth	0.241(2)–0.355(2)	0.69(2)	0.703(17)<br>0.706(17)

III. Calculations

Geometry optimizations were performed at the DFT level of theory with help of hybrid exchange-correlation functional PBE0.[3] The lithium and rubidium atoms were described by the def2-TZVP basis sets (combined with effective core potential for Rb), whereas atoms of organic ligands (C, H) were represented by correlation-consistent basis sets of double-ζ quality (cc-pVDZ). After a number of pilot calculations, this combination of basis sets was found to be a good compromise between accuracy and computational efforts. All calculated structures were tested to be local minima (ground state structures; no imaginary frequencies) or saddle points (transition states; only 1 unique imaginary frequency corresponding to the aimed transformation) on the corresponding potential energy surface, as determined by calculation of the full Hessian matrix followed by estimation of frequencies in the harmonic approximation. The nature of each transition state was additionally traced through the IRC (intrinsic reaction coordinate) technique in both directions (forward and backward).

Energetic evaluation was performed with help of recently developed double-hybrid functional xDH-PBE0[4] for PBE0-optimized geometries. The same combination of basis sets was used. This approach was found to provide accuracy in energetics close to the CCSD(T) level of theory.[5] All calculations were carried out with help of Firefly (version 8.1.0) program package.[6]

Optimized geometries were then used for calculations of NMR parameters. All atoms were described by all-electron Slater-type basis sets of triple-ζ quality (TZ2P). Relativistic ZORA DFT protocol was used for calculations of ^{7}Li NMR parameters with ADF code[7] (NMR module for the NMR properties[8]). NMR chemical shifts were computed at the PBE0/TZ2P/ZORA level of theory.

Table S3. Cartesian coordinates for Li_3Rb_3 optimized at the PBE0/def2-TZVP(Rb,Li)//cc-pVDZ(C,H) level of theory.

Rb	-1.123427134	3.357805886	-0.737478633
Li	-0.047572272	0.156417645	-0.043119550
Li	1.328382268	-2.438660170	-1.185922729
C	-1.195230159	-1.740270238	-0.219716252
C	-1.902467516	-0.897862043	0.666634619
C	-2.311265240	0.253412260	-0.051551249
C	-1.865742317	0.106617437	-1.396240965
C	-1.185528464	-1.130693093	-1.493758535
C	-0.964789318	-1.864838389	-2.714396819
C	-0.467174183	-3.183391490	-2.466324572
H	-0.264486360	-3.848327754	-3.312070462
C	-0.427839912	-3.790404753	-1.148775357
H	-0.168086376	-4.852358021	-1.103906772
C	-0.884843342	-3.123268253	0.029950845
C	-1.268815831	-3.528721967	1.346093057
H	-1.102385563	-4.561806213	1.665633915
C	-2.055701522	-2.689826918	2.231586986
H	-2.448801883	-3.171988607	3.132483252
C	-2.515370388	-1.381109080	1.880366727
C	-3.606740415	-0.553060539	2.294749385
H	-4.211857622	-0.853514181	3.157719210
C	-4.055519165	0.599420903	1.550220120
H	-4.984951255	1.071570489	1.889958787
C	-3.469690376	1.039310748	0.321036433
C	-3.969894735	1.823796663	-0.770627102
H	-4.862224007	2.441661479	-0.616723034
C	-3.511045234	1.682358488	-2.127852963
H	-4.089500731	2.205352425	-2.898012099
C	-2.509464022	0.737219229	-2.528882148
C	-2.259247232	0.042659590	-3.754325084
H	-2.761240148	0.385858027	-4.666686987
C	-1.524919212	-1.195419549	-3.848363863
H	-1.501319757	-1.685683918	-4.828303044
Li	0.196645344	-2.090698272	2.170151329
C	1.954019507	-0.483978338	0.689511751
C	2.147053394	0.203795944	-0.528805646
C	1.714718801	1.542063203	-0.353491678
C	1.270095563	1.680360883	0.993208885
C	1.429720441	0.427527974	1.632135027
C	1.522805507	0.224391205	3.056124855
C	1.916210449	-1.111667067	3.384555557
H	2.032174101	-1.393528023	4.436071668
C	2.401548810	-2.067458506	2.406568730
H	2.811788955	-3.004492056	2.795117540
C	2.534190963	-1.759414796	1.017046648
C	3.244765189	-2.323517080	-0.087851959

H	3.748450880	-3.288018641	0.026427305
C	3.505113172	-1.591763583	-1.313497214
H	4.213794113	-2.051212357	-2.010143246
C	3.073685592	-0.246365972	-1.539067452
C	3.526687419	0.829827207	-2.366572990
H	4.298144799	0.639853220	-3.121499205
C	3.127095261	2.202961754	-2.169869044
H	3.646658587	2.955961474	-2.774036949
C	2.242991661	2.642608943	-1.134163106
C	2.054197575	3.909024112	-0.487940256
H	2.435890573	4.815692502	-0.971718959
C	1.594387500	4.048448174	0.869606528
H	1.661770117	5.050099602	1.309430364
C	1.283527320	2.933840335	1.717216091
C	1.332305744	2.744617588	3.134466792
H	1.425243130	3.625298555	3.780768372
C	1.444506911	1.457884057	3.777129896
H	1.587152861	1.455923778	4.863740141
Rb	-4.180720980	-1.706976956	-1.166544627
Rb	4.210405688	1.651506360	1.174723429
Rb	0.797226123	0.706337483	-3.628977030
Rb	-1.497037117	1.413517348	3.150787698

Table S4. Cartesian coordinates for $LiRb_5$ optimized at the PBE0/def2-TZVP(Rb,Li)//cc-pVDZ(C,H) level of theory.

Rb	-1.108504765	3.309791622	-0.722443306
Li	0.038648781	-0.097991621	0.015642663
Rb	1.644990128	-3.066238161	-1.419784773
C	-1.402666391	-1.950444175	-0.279087728
C	-2.029222371	-1.058993598	0.638295460
C	-2.314268325	0.153727910	-0.051807393
C	-1.864018354	0.011925166	-1.395379734
C	-1.301261669	-1.288593822	-1.536085945
C	-1.263524072	-1.999812653	-2.802991085
C	-1.168150815	-3.418379618	-2.653461770
H	-1.196411229	-4.046714723	-3.551196198
C	-1.271512064	-4.085380425	-1.387417074
H	-1.371652446	-5.176858127	-1.406235309
C	-1.479431703	-3.396451461	-0.152056120
C	-2.091098996	-3.826434208	1.066305164
H	-2.249651436	-4.899218379	1.226605505
C	-2.721854580	-2.928500424	1.990498944
H	-3.317549119	-3.377860314	2.793608167
C	-2.800052352	-1.516069385	1.782678346
C	-3.748407333	-0.552718232	2.247834937

H	-4.418607618	-0.826811696	3.071004253
C	-4.034400546	0.669987474	1.553314823
H	-4.903408067	1.244879685	1.893982415
C	-3.399017421	1.042985683	0.328131056
C	-3.845991379	1.879794664	-0.741520087
H	-4.700240548	2.545070558	-0.570194509
C	-3.390963170	1.738299205	-2.094889665
H	-3.929452348	2.304654188	-2.863678789
C	-2.448367929	0.745241600	-2.505968561
C	-2.250538486	0.108333182	-3.770735546
H	-2.706784193	0.552725910	-4.662991787
C	-1.685300434	-1.202663692	-3.912261661
H	-1.749825932	-1.669044697	-4.902267041
Rb	0.282845432	-2.640025664	2.650939950
C	2.235406656	-0.490212123	0.797028620
C	2.338767429	0.174658995	-0.458268405
C	1.776855323	1.475137846	-0.314941602
C	1.325863468	1.614480518	1.028584630
C	1.609313936	0.399861401	1.716018956
C	1.831638099	0.338452293	3.151122890
C	2.594971518	-0.799872842	3.557629819
H	2.865774957	-0.903227636	4.614838834
C	3.225424421	-1.696813470	2.631796149
H	3.934439018	-2.422902532	3.046223989
C	3.151371247	-1.539258045	1.212714713
C	4.045436556	-1.953135843	0.176517403
H	4.813429929	-2.700047175	0.409263988
C	4.147939659	-1.284639614	-1.089101761
H	4.987246061	-1.567165474	-1.735263765
C	3.366704230	-0.138817121	-1.436958139
C	3.628392778	0.933781882	-2.345305338
H	4.429650876	0.816659841	-3.084220767
C	3.061115589	2.243962436	-2.201676455
H	3.468304942	3.036375156	-2.840342765
C	2.180508578	2.604775494	-1.134729169
C	1.921959532	3.870096206	-0.521509857
H	2.247851143	4.782152024	-1.034954517
C	1.469307129	4.010789079	0.832549395
H	1.481420245	5.020387583	1.259492029
C	1.232173405	2.899800391	1.700352513
C	1.282781034	2.798588766	3.125513257
H	1.281976302	3.719379270	3.720316526
C	1.569353903	1.575421413	3.818266932
H	1.767399904	1.647139423	4.894089617
Rb	-4.242332229	-1.813947675	-1.244719567
Rb	4.320054809	1.609172282	1.282760361
Rb	0.785504735	0.610483124	-3.509544160
Rb	-1.421898906	1.300978507	3.084072406

Table S5. Absolute energies (in Hartree) for Li_3Rb_3 and $LiRb_5$ products calculated at the PBE0/def2-TZVP(Rb,Li)//cc-pVDZ(C,H) level of theory.

Compound	Energy
Li_3Rb_3	-1677.8449897165
$LiRb_5$	-1711.0076952272

Migration of the Li^+ ion

In order to elucidate the migration mechanism of the lithium cation from the position between two 6-membered rings to the center of the sandwich (between two 5-membered rings), we turned to consideration of the parent anionic 5-metal systems ("outside" cations are excluded). Only two systems were considered, namely, α- and β-isomers of Li_3Rb_2, which finally lead to the experimentally observed Li_3Rb_3 compound. In Fig. S7, possible migration pathways resulting in the target 6-metal system are presented. The migration process is schematically shown in Fig. S8.

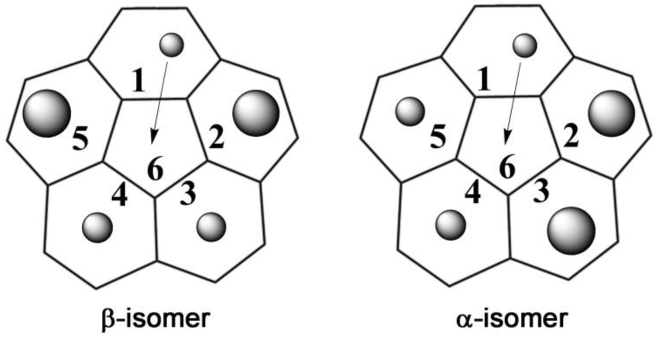

Figure S7. Schematic representation of α- and β-isomers of Li_3Rb_2 along with possible migration pathways, which result in the same target 6-metal product.

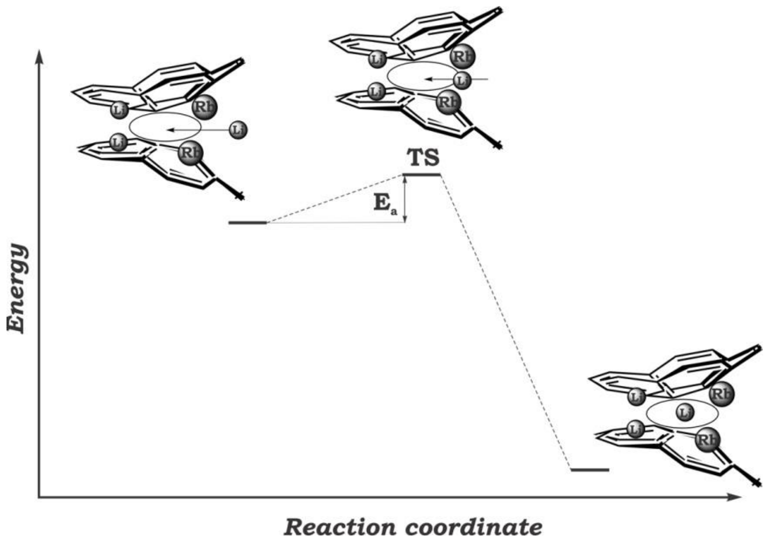

Figure S8. Schematic representation of internal lithium migration for Li_3Rb_2 system.

Table S6. Cartesian coordinates for the parent α-Li_3Rb_2 sandwich optimized at the PBE0/def2-TZVP(Rb,Li)//cc-pVDZ(C,H) level of theory.

Rb	-1.052962128	3.180138736	-0.726945546
Li	1.204673876	-2.315115954	-1.029926716
C	-1.164945199	-1.773358977	-0.280409798
C	-1.748013891	-0.872720672	0.628547373
C	-2.142785844	0.276713011	-0.085048538
C	-1.807552196	0.080843513	-1.446893224
C	-1.201142254	-1.189039743	-1.558182517
C	-1.053512814	-1.949803090	-2.769324395
C	-0.549574284	-3.264858879	-2.509850440
H	-0.346317026	-3.940316176	-3.348367849
C	-0.462377538	-3.847516290	-1.183652965
H	-0.183400073	-4.904527996	-1.126982906
C	-0.877586088	-3.151060357	-0.002388194
C	-1.197332977	-3.497133118	1.351362079
H	-1.022890186	-4.517064208	1.707020514
C	-1.836659103	-2.584403730	2.276862565
H	-2.099369297	-2.985753321	3.260386418
C	-2.189631613	-1.235285246	1.946188190
C	-2.985133371	-0.199284786	2.537407744
H	-3.375945788	-0.320987946	3.552677805
C	-3.483599416	0.929485221	1.773736558
H	-4.227966512	1.560077638	2.273298591
C	-3.199804079	1.140376993	0.386230739
C	-3.839012414	1.844789752	-0.684021409

H	-4.681135389	2.513169135	-0.466607333
C	-3.513522479	1.635556603	-2.084225046
H	-4.167984859	2.124017542	-2.816791198
C	-2.541588045	0.683310382	-2.541289878
C	-2.362587413	-0.029735926	-3.771505545
H	-2.898957313	0.311466899	-4.665995401
C	-1.629248867	-1.275659864	-3.892605016
H	-1.638570836	-1.762572805	-4.875076961
Li	0.205897872	-1.866954699	1.848421222
C	1.929799394	-0.513733152	0.631525843
C	2.151979829	0.189471956	-0.569819844
C	1.711099276	1.521856891	-0.382485546
C	1.211874109	1.625241770	0.933995759
C	1.348023885	0.372172084	1.556063773
C	1.220846231	0.124271432	2.963583575
C	1.701763123	-1.178356674	3.319081466
H	1.661636109	-1.506258301	4.362162462
C	2.338965401	-2.076008151	2.377731471
H	2.733746745	-3.013015102	2.782238389
C	2.530483649	-1.768261708	0.991283033
C	3.281589046	-2.323324233	-0.096386862
H	3.778370334	-3.291801941	0.019454068
C	3.597854837	-1.562619522	-1.290955975
H	4.325593057	-2.015485768	-1.973953877
C	3.165966188	-0.215890016	-1.514516651
C	3.635801352	0.880962074	-2.305768146
H	4.430524712	0.715714136	-3.043577949
C	3.200404790	2.252416066	-2.108456331
H	3.736036174	3.022524968	-2.677228503
C	2.279860225	2.654713603	-1.083344602
C	2.049698917	3.890137019	-0.394402619
H	2.465617064	4.816166893	-0.810945319
C	1.426796187	3.990658182	0.911830447
H	1.388000213	4.987554057	1.366975067
C	1.022718981	2.846331716	1.669441508
C	0.668589875	2.593450023	3.033622274
H	0.450775385	3.435539639	3.699924004
C	0.762853889	1.288696303	3.661427918
H	0.590850786	1.247383534	4.741716572
K	-4.017608309	-1.416641906	-1.171848681
K	4.060010172	1.490866970	0.920238710
Rb	0.741406816	0.619505171	-3.366101425
Li	-1.015623758	0.920480828	2.297602809

Table S7. Cartesian coordinates for the transition state in α-Li_3Rb_2 optimized at the PBE0/def2-TZVP(Rb,Li)//cc-pVDZ(C,H) level of theory.

Rb	-1.140454446	3.358618967	-0.421794568
Li	1.001430370	-2.410998325	-1.234760304
C	-1.285227847	-1.858155924	-0.490013278
C	-1.828143060	-0.841341568	0.299091829
C	-2.170722343	0.246783562	-0.536479906
C	-1.876675231	-0.137307581	-1.868616418
C	-1.324628951	-1.432575523	-1.827606140
C	-1.125511369	-2.309076123	-2.940251068
C	-0.637381428	-3.586855078	-2.516254208
H	-0.414591326	-4.356717657	-3.262685797
C	-0.547870351	-4.002518156	-1.122341908
H	-0.238094326	-5.036352771	-0.939938899
C	-0.936764820	-3.165302208	-0.031863665
C	-1.164930529	-3.299350312	1.383534512
H	-1.007351725	-4.269062807	1.868907390
C	-1.762398066	-2.254623673	2.199235495
H	-2.010710974	-2.521320955	3.233095570
C	-2.151749157	-0.969075197	1.689197376
C	-2.906183938	0.162907367	2.141690324
H	-3.207122831	0.231341530	3.191771953
C	-3.475304291	1.137770242	1.239176964
H	-4.196165443	1.839675696	1.676674789
C	-3.225129476	1.166240029	-0.171021660
C	-3.820658010	1.781053423	-1.318012610
H	-4.638966593	2.500896134	-1.184497512
C	-3.504386582	1.409546681	-2.683722289
H	-4.126354953	1.857783509	-3.467788531
C	-2.600916606	0.344198579	-3.022068533
C	-2.420873312	-0.507513014	-4.157018105
H	-2.921170730	-0.253625976	-5.100598221
C	-1.646373366	-1.733150216	-4.149157526
H	-1.629523069	-2.311122080	-5.080028928
Li	0.447470835	-1.999030269	2.233692347
C	1.765753637	-0.464702460	0.883205375
C	1.783453309	0.321440373	-0.299862036
C	1.507614973	1.661097884	0.068683801
C	1.323700660	1.702552684	1.474577223
C	1.503295413	0.397677652	1.977315504
C	1.732630318	0.086394496	3.357154702
C	2.185570285	-1.259478485	3.527868889
H	2.405389978	-1.634662345	4.532514837
C	2.582032408	-2.108381383	2.419050105
H	3.070271056	-3.055428142	2.670022249
C	2.460506504	-1.725332525	1.041093763
C	2.999367883	-2.214171612	-0.186061647
H	3.556796252	-3.156501896	-0.196975664

C	3.067733746	-1.391257025	-1.392611588
H	3.735603847	-1.751992702	-2.183724157
C	2.620313620	-0.030626790	-1.428896846
C	3.025840783	1.111398846	-2.189532285
H	3.712383408	0.973359065	-3.036013425
C	2.632561042	2.472317818	-1.880479049
H	3.102827974	3.266183561	-2.473261439
C	1.949723608	2.822365474	-0.672986807
C	1.884025709	4.024868583	0.105081814
H	2.204048333	4.970264754	-0.351558408
C	1.553778537	4.062994950	1.505899223
H	1.631666130	5.035748703	2.005357509
C	1.357419324	2.881799868	2.288967049
C	1.451890327	2.568030176	3.676773074
H	1.489630927	3.385147094	4.406316995
C	1.631882541	1.245522417	4.184034806
H	1.774730297	1.143602068	5.265415443
K	-4.221295291	-1.375894491	-1.332491657
K	4.104735727	1.171428531	0.868183845
Rb	0.460541736	0.571014730	-3.638813645
Li	-0.500678631	0.510706294	1.189961614

Table S8. Cartesian coordinates for the product of migration in α-Li_3Rb_2 optimized at the PBE0/def2-TZVP(Rb,Li)//cc-pVDZ(C,H) level of theory.

Rb	-1.293253532	3.256159923	-0.043047570
Li	1.319815802	-2.425086042	-1.166092844
C	-1.212451256	-1.753119043	-0.243710431
C	-1.908944197	-0.900592361	0.637895370
C	-2.311328508	0.244559283	-0.082942814
C	-1.849948284	0.105803739	-1.424630525
C	-1.183538901	-1.143871247	-1.516381395
C	-0.959255373	-1.891966993	-2.731515667
C	-0.452624919	-3.201646806	-2.461934201
H	-0.248867491	-3.878164156	-3.299270506
C	-0.412301614	-3.800339686	-1.129713967
H	-0.134907014	-4.857841423	-1.074438371
C	-0.879220883	-3.123765292	0.036377360
C	-1.235355339	-3.487385318	1.376108654
H	-1.039107872	-4.504819949	1.728725632
C	-1.999572556	-2.628249973	2.260542344
H	-2.334014485	-3.071748599	3.204460987
C	-2.435822006	-1.315125179	1.905189083
C	-3.425254589	-0.404389924	2.390134800
H	-3.913676958	-0.601611051	3.350365495

C	-3.921146711	0.686831120	1.621379141
H	-4.780103175	1.230355493	2.033743791
C	-3.454434766	1.030583246	0.312810701
C	-4.003942995	1.774918334	-0.780838160
H	-4.890248325	2.400760468	-0.616989860
C	-3.545158552	1.643425459	-2.142970707
H	-4.146987738	2.145484987	-2.910524444
C	-2.534665182	0.707068280	-2.558436597
C	-2.302361124	-0.005315766	-3.772222134
H	-2.819053281	0.318846528	-4.684590408
C	-1.521157824	-1.230102660	-3.870411605
H	-1.499986019	-1.725238376	-4.848368683
Li	0.098917971	-1.854243205	2.200774184
C	1.966868549	-0.486981605	0.677319173
C	2.131905942	0.191105361	-0.553134851
C	1.720092412	1.541377163	-0.367033028
C	1.321111393	1.680507128	0.990798583
C	1.462806864	0.429704293	1.623240981
C	1.436919650	0.209208194	3.037553531
C	1.842880590	-1.118682798	3.376320459
H	1.882691945	-1.422461291	4.427702898
C	2.357445956	-2.067145534	2.406828816
H	2.733874577	-3.016319332	2.801418170
C	2.528620814	-1.767591981	1.016810101
C	3.238679319	-2.339447961	-0.082146026
H	3.738351823	-3.306514182	0.032116854
C	3.516032044	-1.597853021	-1.310530775
H	4.241174280	-2.054006995	-1.993781116
C	3.088827872	-0.254348343	-1.547249324
C	3.555605907	0.827167559	-2.362878287
H	4.335663170	0.640013974	-3.110374770
C	3.172982199	2.217908230	-2.148269241
H	3.710682910	2.969647586	-2.739583305
C	2.280675651	2.651953714	-1.121726547
C	2.088362521	3.909488650	-0.452415961
H	2.481398647	4.820170872	-0.921533795
C	1.618922104	4.039133169	0.903897101
H	1.657163145	5.042270433	1.346383942
C	1.278059070	2.915843308	1.724880751
C	1.150478645	2.700683737	3.132991880
H	1.098780238	3.571562820	3.797728979
C	1.203952162	1.422455717	3.756794239
H	1.159367088	1.398438947	4.850732057
K	-4.001696396	-1.506844844	-1.388494900
K	4.164279131	1.504166456	0.765250161
Rb	0.673633394	0.860213968	-3.424665179
Li	-0.049342349	0.164438555	-0.049047041

Table S9. Absolute energies (in Hartree) for parent compound, transition state and product of insertion for α-Li_3Rb_2 calculated at different levels of theory.

Compound	Energy	
	PBE0	xDH-PBE0
α-Li_3Rb_3-parent	-2805.0573976286	-2803.8114014895
α-Li_3Rb_3-TS	-2805.0398094444	-2803.7867318421
α-Li_3Rb_3-product	-2805.0563548444	-2803.8051350083

β-Li_3Rb_2 system

In the case of the β-Li_3Rb_2 system, we were not able to localize the parent geometrical configurations, in which the space between two 5-membered rings is empty. All attempts to perform geometry optimization for such system unambiguously resulted in the product of insertion of the lithium cation to the center of sandwich. Different thresholds for gradient convergence were tested with the same final result. On the basis of these extensive calculations, we concluded that the insertion process proceeds in a barrierless manner.

Table S10. Cartesian coordinates for the product of migration in β-Li_3Rb_2 optimized at the PBE0/def2-TZVP(Rb,Li)//cc-pVDZ(C,H) level of theory.

```
--------------------------------------------------------------------
Rb      -0.802533692      3.079333172     -1.439204189
Rb       1.633130134     -2.568504555     -2.046617307
C       -1.244095658     -1.857341420     -0.391624079
C       -1.756572728     -0.934450805      0.545996306
C       -2.147761767      0.230810841     -0.148735210
C       -1.879984558      0.033558734     -1.527983887
C       -1.317015588     -1.266181433     -1.679308197
C       -1.471583397     -2.067208449     -2.877408027
C       -1.290531500     -3.463720218     -2.636813568
H       -1.421331398     -4.165461734     -3.470505589
C       -1.073526962     -4.040956710     -1.332962596
H       -1.045531584     -5.134958026     -1.271964873
C       -1.126505882     -3.271661613     -0.124700422
C       -1.344637741     -3.580137700      1.250012518
H       -1.300484173     -4.623946488      1.579002464
C       -1.831254764     -2.613610781      2.226134202
H       -2.084450736     -3.000666298      3.218108299
C       -2.145300251     -1.259787257      1.893726793
C       -2.880754224     -0.198241575      2.506026852
H       -3.251470863     -0.316170436      3.529013196
C       -3.328071998      0.982649571      1.778822458
```

H	-4.026474327	1.642492143	2.304667205
C	-3.065377973	1.204407071	0.395129010
C	-3.680874246	1.991486011	-0.632776167
H	-4.443174100	2.730059738	-0.359291172
C	-3.524499698	1.702023752	-2.036682399
H	-4.188126990	2.238158341	-2.727147074
C	-2.663995894	0.687225911	-2.557062877
C	-2.658340825	-0.038467825	-3.792594730
H	-3.225330398	0.359008000	-4.643113253
C	-2.094683228	-1.340703056	-3.944075128
H	-2.268739431	-1.848286559	-4.900496273
Li	0.301937742	-2.160207498	1.919824646
C	2.131949895	-0.512340753	0.605944500
C	2.414994099	0.206412015	-0.584397554
C	1.856726353	1.509568101	-0.446960860
C	1.234509090	1.580844842	0.825833346
C	1.406942937	0.336605742	1.470434354
C	1.204270787	0.103182605	2.876680502
C	1.720329517	-1.165806821	3.284236930
H	1.647347290	-1.468901079	4.333371514
C	2.511156687	-2.021534444	2.408868321
H	2.995503938	-2.883668352	2.879804149
C	2.836661756	-1.687918495	1.061437632
C	3.870901942	-2.078688559	0.148853827
H	4.521324028	-2.923090183	0.404217222
C	4.256448661	-1.275627389	-0.985275152
H	5.179802160	-1.565384228	-1.503038736
C	3.591047723	-0.075546527	-1.383350938
C	4.014445273	1.057486813	-2.151721264
H	4.900914235	0.963211399	-2.790460094
C	3.452850312	2.362345188	-2.015876081
H	3.948242378	3.174767663	-2.561175339
C	2.404370965	2.684994501	-1.094061478
C	2.029323748	3.900391328	-0.443549792
H	2.420253542	4.850041170	-0.830658472
C	1.270748085	3.963870586	0.781473636
H	1.131021035	4.954615300	1.228995752
C	0.905396478	2.797572981	1.529925287
C	0.536868640	2.552116958	2.885096125
H	0.282518073	3.397724070	3.533147539
C	0.677005452	1.255936680	3.536599521
H	0.487667738	1.224102783	4.614118818
K	-3.988118075	-1.682341780	-1.362082067
K	4.145341049	1.546958576	1.031259066
Li	0.126672720	-0.012547992	-0.397953904
Li	-1.126938735	1.123323092	2.278999146

Table S11. Absolute energies (in Hartree) for the product of insertion for β-Li_3Rb_2 calculated at different levels of theory.

Compound	Energy	
	PBE0	xDH-PBE0
β-Li_3Rb_3-product	-2805.0643732285	-2803.8131107291

IV. References

[1] N. V. Kozhemyakina, J. Nuss and M. Jansen, *Z. Anorg. Allg. Chem.*, 2009, **635**, 1355.

[2] L. T. Scott, P.-C. Cheng, M. M. Hashemi, M. S. Bratcher, D. T. Meyer and H. B. Warren, *J. Am. Chem. Soc.*, 1997, **119**, 10963.

[3] (a) J. P. Perdew, K. Burke and M. Ernzerhof, *Phys. Rev. Lett.*, 1996, **77**, 3865; (b) J. P. Perdew, K. Burke and M. Ernzerhof, *Phys. Rev. Lett.*, 1997, **78**, 1396.

[4] I. Y. Zhang, N. Q. Su, E. A. G. Brémond, C. Adamo and X. Xu, *J. Chem. Phys.*, 2012, **136**, 174103.

[5] N. Q. Su, C. Adamo and X. Xu, *J. Chem. Phys.*, 2013, **139**, 174106.

[6] A. A. Granovsky, *Firefly, version 8.1.0*, http://classic.chem.msu.su/gran/gamess/index.html

[7] (a) C. F. Guerra, J. Snijders, G. te Velde and E. Baerends, *Theor. Chem. Acc*., 1998, **99**, 391; (b) G. te Velde, F. Bickelhaupt, E. Baerends, C. Guerra, S. Van Gisbergen, J. Snijders and T. Ziegler, *J. Comput. Chem*., 2001, **22**, 931.

[8] (a) S. Wolff, T. Ziegler, E. van Lenthe and E. Baerends, *J. Chem. Phys*., **1999**, *110*, 7689; (b) G. Schreckenbach and T. Ziegler, *J. Phys. Chem*., 1995, **99**, 606.
